# Comparative analysis of TNF-α, TLR-2, and TLR-4 in GCF before and after periodontal therapy

**DOI:** 10.1590/1807-3107bor-2026.vol40.012

**Published:** 2026-05-08

**Authors:** Mithat TERZI, Fatma ALTIPARMAK, Fatma Ucan YARKAC

**Affiliations:** (a) Hisar Intercontinental Hospital, Istanbul, Turkey.; (b) Karamanoglu Mehmetbey University, Faculty of Dentistry, Department of Periodontology, Karaman, Turkey.; (c) Necmettin Erbakan Üniversitesi, Faculty of Dentistry, Department of Periodontology, Konya, Turkey.

**Keywords:** Biomarkers, Gingival Crevicular Fluid, Periodontal Diseases, Periodontitis, Root Planing

## Abstract

Periodontitis is a chronic inflammatory disease characterized by progressive destruction of tooth-supporting tissues, primarily driven by a dysregulated host immune response to subgingival biofilms. Toll-like receptors (TLR-2 and TLR-4) and tumor necrosis factor-alpha (TNF-α) are key mediators of immune signaling and tissue breakdown, making them potential biomarkers of periodontal disease activity. This prospective, non-randomized interventional study evaluated TLR-2, TLR-4, and TNF-α levels in gingival crevicular fluid (GCF) for periodontal disease monitoring and assessed the effects of nonsurgical periodontal therapy (NSPT) on clinical parameters. Forty systemically healthy individuals were enrolled and assigned to either periodontitis or healthy groups. Clinical indices—gingival index, plaque index, clinical attachment loss, and probing pocket depth—were recorded at baseline and 8 weeks after NSPT. GCF samples were analyzed using enzyme-linked immunosorbent assay to determine biomarker concentrations. At baseline, TNF-α and TLR-2 levels were significantly higher in periodontitis patients compared with healthy controls (p < 0.001). Following therapy, marked clinical improvements were observed, accompanied by significant reductions in TNF-α and TLR-4 levels (p < 0.05), while TLR-2 levels remained stable. receiver operating characteristic analysis demonstrated that TNF-α and TLR-2 reliably distinguished disease from health. In conclusion, NSPT effectively improved clinical outcomes and reduced TNF-α and TLR-4 levels in GCF, reflecting a decreased local inflammatory burden. The persistence of TLR-2 expression post-treatment suggests a stable role in immune surveillance rather than acute inflammatory response.

## Introduction

Periodontitis is a chronic inflammatory disorder characterized by progressive degradation of tooth-supporting structures, primarily resulting from an altered immune response to bacterial biofilms.^1^ Although biofilms initiate the disease, its severity and progression are largely determined by the host’s immune-inflammatory mechanisms.^2^ The persistent interaction between bacterial pathogens and the immune system sustains inflammation, ultimately leading to the breakdown of periodontal tissues.^3^

Toll-like receptors (TLRs), a key subset of pattern recognition receptors, play a central role in the host’s early defense mechanisms against periodontal pathogens. These membrane-bound proteins recognize conserved microbial structures, known as pathogen-associated molecular patterns, and translate these signals into cellular immune responses. Through this recognition, TLRs activate downstream signaling cascades, triggering proinflammatory pathways that influence the onset and severity of periodontal inflammation.^4^ Among these receptors, TLR-2 and TLR-4 are particularly important in periodontitis due to their ability to detect bacterial lipoproteins and lipopolysaccharides, respectively.^5^ Upon activation, these receptors stimulate the production of inflammatory mediators such as tumor necrosis factor-alpha (TNF-α), interleukin (IL)-1β, and IL-6, all of which contribute to periodontal tissue destruction.^6^ Furthermore, genetic variations in TLR-4 have been linked to increased susceptibility to chronic periodontitis, highlighting its role in modulating host–microbial interactions.^5^

TNF-α is a central proinflammatory cytokine downstream of TLR signaling and plays a critical role in the pathogenesis of periodontal disease.^7^ It promotes osteoclast differentiation, enhances matrix metalloproteinase activity, and contributes to extracellular matrix degradation, leading to connective tissue destruction and alveolar bone loss.^8^ Elevated TNF-α concentrations in gingival crevicular fluid (GCF) have been consistently associated with disease severity, underscoring its potential as a biomarker for periodontal inflammation and progression.^9^ Periodontal therapy primarily aims to reduce microbial challenge, regulate host-mediated inflammation, and restore tissue homeostasis through nonsurgical strategies such as scaling and root planing (SRP). Profiling TNF-α, TLR-2, and TLR-4 levels after therapy may therefore provide valuable insights into immunological modulation and treatment effectiveness.

Correlating these biomarkers with clinical parameters—including gingival index (GI), plaque index (PI), clinical attachment loss (CAL), and probing pocket depth (PPD)—can yield a more comprehensive understanding of the molecular and clinical mechanisms underlying periodontitis. Previous studies have reported increased TLR-2 and TLR-4 expression in gingival tissues^11,12^ as well as altered TLR-related biomarkers in saliva and GCF.^13^ Treatment has been shown to influence these signals, with reductions in TLR-4 activity and improvements in GCF markers.^14^ Similarly, TNF-α has been strongly associated with periodontal status.^15^ However, only a limited number of studies have concurrently evaluated TLRs and inflammatory mediators in GCF and examined their modulation following periodontal therapy.

Therefore, the present study investigates the impact of periodontal treatment on GCF levels of TNF-α, TLR-2, and TLR-4 and explores their relationships with key clinical periodontal parameters. By analyzing these immune-inflammatory markers at baseline and after therapy, this study aims to provide deeper insights into the biological effects of periodontal treatment and its role in modulating inflammatory pathways involved in disease progression.

## Methods

This prospective, non-randomized interventional study included 40 systemically healthy volunteers, comprising 12 males and 28 females aged 20–50 yr. Participants were recruited from patients presenting to the Faculty of Dentistry, Necmettin Erbakan University, Konya, Turkey, between June and December 2021. Ethical approval was obtained from the Non-Interventional Clinical Research Ethics Committee for Pharmaceuticals and Medical Devices of Necmettin Erbakan University (Decision No. 2021/05-63). All methods were performed in accordance with relevant guidelines and regulations, including the 2008 revision of the Declaration of Helsinki. Before enrollment, each participant was informed verbally and in writing about the study objectives, procedures, and potential implications. Written informed consent was obtained from all participants and/or their legal guardians.

### Sample size calculation

In the absence of directly comparable data on pre–post changes in GCF TLR-4 levels following nonsurgical periodontal therapy (NSPT), the sample size was determined using pilot study guidance.^16,17^ A total of 40 systemically healthy individuals were enrolled and assigned to two groups according to periodontal status: Group 1 (periodontally healthy, n = 20) and Group 2 (periodontitis patients, n = 20). All participants completed follow-up. For within-patient comparison in the periodontitis group (paired t test, two-tailed), post hoc analysis indicated an observed standardized mean change (Cohen’s d) of 0.86. With α = 0.05 and n = 20 pairs, the achieved power was 1 − β ≈ 0.96 (G^*^Power 3.1), confirming adequacy of the sample size.

### Study design

**Figure 1:**
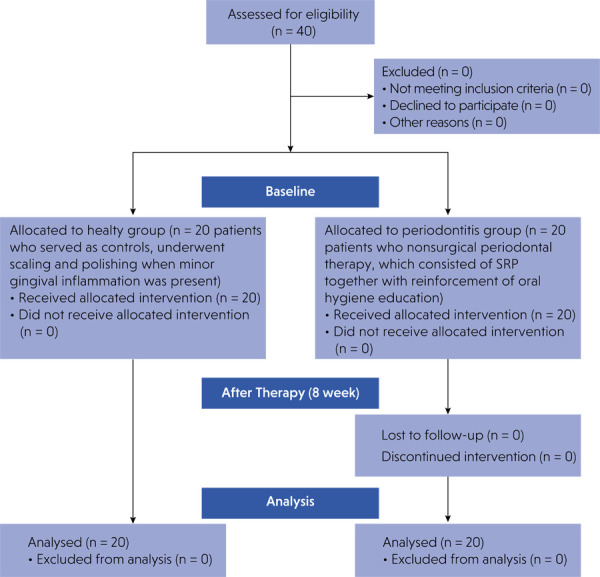
Flow diagram of participants throughout the study

Patients presenting to the Periodontology Clinic were screened for eligibility through detailed medical and dental history reviews, followed by clinical and radiographic examinations. Figure 1 summarizes participant flow throughout the study.

Eligible participants were 20–50 years old with no systemic health problems. Periodontally healthy individuals exhibited no CAL, no gingival inflammation (e.g., redness, swelling, or exudate), probing depths ≤ 3 mm, < 10% bleeding on probing (BOP), and no radiographic evidence of alveolar bone loss. Periodontitis patients were diagnosed according to the 2017 World Workshop on the Classification of Periodontal and Peri-Implant Diseases and Conditions.^18^ They presented with generalized periodontitis, Stage II or III and Grade B, characterized by PPD > 4 mm, CAL ≥ 3 mm, BOP, and radiographically evident bone loss.

Exclusion criteria included immunodeficiency; systemic conditions requiring prophylactic antibiotics before dental procedures; periodontal therapy within the past 3 months; systemic antibiotic or anti-inflammatory drug (NSAIDs or corticosteroids) use within the past 3 months or during follow-up; any history of tobacco use (cigarettes, e-cigarettes, smokeless tobacco, or waterpipe); and pregnancy, breastfeeding, or menopause.

Clinical periodontal assessments were performed at baseline and 8 weeks after treatment in the periodontitis group. A calibrated examiner (F.U.Y.) using a manual periodontal probe conducted all examinations. Calibration was completed on 15 volunteers not included in the main study, assessed in two sessions 24 h apart. The intraclass correlation coefficient for PPD measurements between sessions was 0.94 ± 0.05, confirming excellent intra-examiner reliability. Healthy participants were examined only at baseline.

Periodontal parameters were assessed across the full dentition. PI was evaluated at six sites per tooth based on supragingival plaque accumulation.^19^ GI was visually determined using a 0–3 scale, reflecting the severity of inflammation by gingival color, texture, edema, and spontaneous bleeding.^20^ PPD and CAL were measured at six sites per tooth (mesio-buccal, mid-buccal, disto-buccal, mesio-lingual, mid-lingual, and disto-lingual) and rounded to the nearest millimeter using a UNC-15 periodontal probe (Hu-Friedy, Chicago, Illinois). BOP was recorded after depth measurements and expressed as the percentage of affected sites.

### Nonsurgical periodontal therapy

All participants received detailed oral hygiene instructions, including plaque control techniques such as tooth brushing and interdental cleaning, from the calibrated examiner (F.U.Y.). Patients with periodontitis underwent NSPT, consisting of SRP combined with reinforcement of oral hygiene education. All NSPT procedures and post-treatment oral hygiene reinforcement were performed by a single experienced periodontist (F.A.). No antimicrobial agents were administered.

Phase I therapy in the periodontitis group comprised thorough SRP and oral hygiene instruction reinforcement. GCF samples and clinical parameters were collected at baseline and 8 weeks after treatment (± 3 days). Treatment was scheduled within two weeks of the initial examination, with a maximum variation of four days. Healthy participants (controls) underwent scaling and polishing if minor gingival inflammation was observed, along with oral hygiene reinforcement, performed by the same periodontist (F.A.).

All clinical measurements were performed by the calibrated examiner (F.U.Y.). Clinical and GCF assessments were conducted at baseline and 8 weeks after treatmentin the periodontitis group, while the healthy group was assessed only at baseline.

### Collection and evaluation of GCF

Gingival crevicular fluid samples were collected from the deepest periodontal pocket of two teeth per participant in the periodontitis group, both at baseline and 8 weeks after treatment. In the periodontally healthy group, samples were obtained from the mesiobuccal sulcus of two maxillary anterior teeth (central incisors) with probing depth ≤ 3 mm and no clinical signs of inflammation.

Before collection, supragingival plaque was gently removed with a manual scaler. The selected sites were isolated with cotton rolls and dried using an air syringe to minimize contamination. Sterile Periopaper strips (ProFlow, Amityville, New York) were inserted 1 mm into the gingival sulcus and left in place for 30 s. Strips contaminated with blood were excluded.

All strips collected from each participant were pooled into labeled Eppendorf tubes and immediately stored at −80°C. On the day of analysis, samples were eluted in 100 μL of phosphate-buffered saline, incubated at room temperature for 30 min without agitation, and centrifuged for 10 min. The resulting supernatant was used for subsequent quantification.

Levels of TNF-α, TLR-2, and TLR-4 in GCF were measured using human-specific sandwich enzyme-linked immunosorbent assay kits (Elabscience, Wuhan, China), following the manufacturer’s instructions. Final concentrations were determined by comparing optical density values against standard curves generated during each assay. Kit specifications were as follows: TLR-2, sensitivity 0.19 ng/mL, detection range 0.31–20 ng/mL; TLR-4, sensitivity 3.75 pg/mL, detection range 6.25–400 pg/mL, total assay time 3 h 30 min; TNF-α, sensitivity 4.69 pg/mL, and detection range 7.81–500 pg/mL.

### Statistical analysis

All statistical analyses were performed using SPSS software (version 19.0; IBM Corp., Armonk, USA). Continuous variables were expressed as mean ± standard deviation (SD). The Kolmogorov–Smirnov test was used to assess data normality. Independent-samples t tests were applied to compare the control and periodontitis groups at baseline and at the 8-week follow-up. Paired-samples t tests were used for within-group comparisons across time points. Chi-square tests were applied to analyze categorical variables recorded at baseline and post-treatment.

Pearson correlation analysis was performed to evaluate associations between biomarker concentrations and clinical periodontal parameters. Receiver operating characteristic (ROC) curve analysis was conducted to assess the diagnostic utility of GCF biomarkers in distinguishing periodontitis cases from healthy individuals. Sensitivity and specificity were estimated by calculating the area under the curve (AUC). A p-value < 0.05 was considered statistically significant.

## Results

### Descriptive data of participants

Table 1 summarizes the demographic characteristics of participants, including gender, education level, oral hygiene frequency, dental visits, and age. No significant differences were observed between groups for gender (p = 0.366) or age (p = 0.057). However, educational attainment differed significantly (p < 0.001): The periodontitis group included a higher proportion of participants with only primary school education, whereas the periodontally healthy group had more participants with university-level education. Brushing frequency also varied significantly; a larger percentage of participants in the periodontitis group reported brushing less than or equal once per day (p < 0.001).

**Table 1:** Descriptive data of participants.

Variables	Periodontal healthy	Periodontitis	p-value*
Gender [n (%)]			
Female	15 (75)	13 (65)	0.366
Male	5 (25)	7 (35)	
Education			
Primary school	0 (0)	14 (70)	
High school	8 (40)	3 (15)	0.000^*^
University	12 (80)	3 (15)	
Brushing of teeth			
Once a day	6 (30)	19 (95)	0.000^*^
Twice a day	14 (70)	1 (5)	
Dental visits			
When feeling toothache	7 (35)	5 (25)	0.366
Once a year	13 (65)	15 (75)	
Age (mean SD)	31.65 ± 9.01	36.65 ± 6.96	0.057

All data (except for age) are given as n (%). *p-values in bold indicate statistical significance.

### Clinical parameters

Table 2 presents the clinical periodontal measurements. At baseline, individuals with periodontitis had significantly higher PPD, CAL, PI, and GI values compared with healthy controls (p < 0.001). Following nonsurgical therapy, significant reductions were observed in PPD, PI, and GI in the periodontitis group (p < 0.001). Although the reduction in CAL was less marked, it remained statistically significant (p < 0.001). These findings indicate that the intervention produced measurable improvements in clinical periodontal parameters.

**Table 2:** Specifications of clinical criteria in patients.

Clinical criteria	Periodontal healthy (baseline)	Periodontitis (baseline)	Periodontitis (after therapy)	p-value^*^	p-value**	p-value^3***^
	Mean ± SD	Mean ± SD	Mean ± SD			
PPD	1.48 ± 0.18	3.72 ± 0.65	2.64 ± 0.43	< 0.001^*^	< 0.001^*^	< 0.001^*^
CAL	-	4.04 ± 0.63	2.92 ± 0.45			< 0.001^*^
PI	0.24 ± 0.23	2.33 ± 0.72	1.22 ± 0.41	< 0.001^*^	< 0.001^*^	< 0.001^*^
GI	0.11 ± 0.11	2.02 ± 0.34	1.17 ± 0.37	< 0.001^*^	< 0.001^*^	< 0.001^*^

- not applicable (CAL not measured in the healthy group); CAL: clinical attachment loss; GI: gingival index; PI: plaque index; PPD: probing pocket depth. Data are presented as mean ± standard deviation (SD). The healthy group was assessed at baseline only. p-value*: Periodontal healthy (baseline) versus periodontitis (baseline), independent-samples t test. p-value**: = Periodontal healthy (baseline) versus periodontitis (after therapy), independent-samples; t: test; p-value***: Periodontitis (baseline) versus periodontitis (after therapy), paired t test.

### Cytokine levels in GCF

As shown in Table 3, baseline concentrations of TNF-α, TLR-2, and TLR-4 were elevated in the periodontitis group compared with controls, with statistically significant differences for TNF-α and TLR-2 (p < 0.001). After nonsurgical therapy, significant reductions were observed in TNF-α and TLR-4 (p < 0.05), whereas TLR-2 levels did not differ significantly within the periodontitis group (p = 0.155).

**Table 3:** Comparison of the TNF-α, TLR-2 and TLR-4 concentrations in GCF at baseline and after therapy.

Biomarkers	Periodontal healthy (Baseline)	Periodontitis (Baseline)	Periodontitis (After therapy)	p-value*	p-value**	p-value***
	Mean ± SD	Mean ± SD	Mean ± SD			
TNF-α (pg/mL)	7.03 ± 0.41	9.67 ± 2.76	6.10 ± 2.35	< 0.001^*^	0.090	0.001
TLR-2 (ng/mL)	0.34 ± 0.11	0.60 ±0.27	0.49 ± 0.12	< 0.001^*^	0.001	0.155
TLR-4 (pg/mL)	580.10 ± 485.38	883.56 ± 487.13	457.74 ± 391.96	0.056	0.386	0.001

TLR-2, toll-like receptor-2; TLR-4, toll-like receptor-4; TNF-α, tumor necrosis factor alpha. Data are presented as mean ± standard deviation (SD). The healthy group was assessed at baseline only. -value*: Periodontal healthy (baseline) versus periodontitis (baseline), independent-samples t test. p-value**: = Periodontal healthy (baseline) versus periodontitis (after therapy), independent-samples; t: test; p-value***: Periodontitis (baseline) versus periodontitis (after therapy), paired t test.

### ROC Curve Analysis

**Figure 2:**
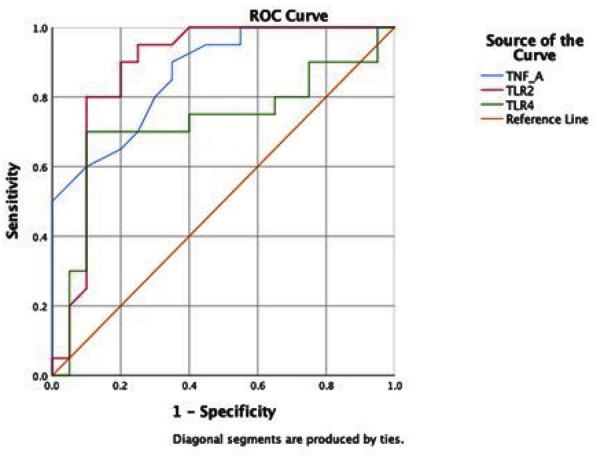
ROC curve

Receiver operating characteristic curve analysis was used to assess the diagnostic performance of TNF-α, TLR-2, and TLR-4 in distinguishing periodontal health from disease (Figure 2). TLR-2 demonstrated the highest diagnostic accuracy (AUC = 0.883), followed by TNF-α (AUC = 0.867), both indicating excellent discriminative ability. TLR-4 showed only moderate diagnostic capacity (AUC = 0.723). These results suggest that TLR-2 and TNF-α are reliable biomarkers for periodontal disease detection, whereas TLR-4 has limited diagnostic utility.

### Correlations

Table 4 presents correlations between clinical parameters, biomarker levels, and age. Age correlated strongly with PI (r = 0.832, p < 0.001), GI (r = 0.798, p < 0.001), and PPD (r = 0.688, p < 0.001), and moderately with TLR-2 (r = 0.445, p = 0.004) but not with TNF-α (r = 0.271, p = 0.091) or TLR-4 (r = −0.108, p = 0.507).

**Table 4:** Pearson correlation.

	Age	PI	GI	PPD	TNF-α	TLR-2	TLR-4
Age	1	0.832^*^	0.798^*^	0.688^*^	0.271	0.445^*^	−0.108
PI		1	0.898^*^	0.806^*^	0.427^*^	0.469^*^	0.091
GI			1	0.922^*^	0.417^*^	0.472^*^	0.023
PPD				1	0.369^**^	0.371^**^	0.014
TNF- α					1	−0.012	0.016
TLR-2						1	0.400^**^
TLR-4							1

*Correlation is significant at the 0.01 level (two-tailed). **Correlation is significant at the 0.05 level (two -tailed).

Clinical parameters were positively correlated with biomarker levels: PI with TNF-α (r = 0.427, p = 0.006) and TLR-2 (r = 0.469, p = 0.002); GI with TNF-α (r = 0.417, p = 0.007) and TLR-2 (r = 0.472, p = 0.002); and PPD with TNF-α (r = 0.369, p = 0.019) and TLR-2 (r = 0.371, p = 0.018)

**Figure 3:**
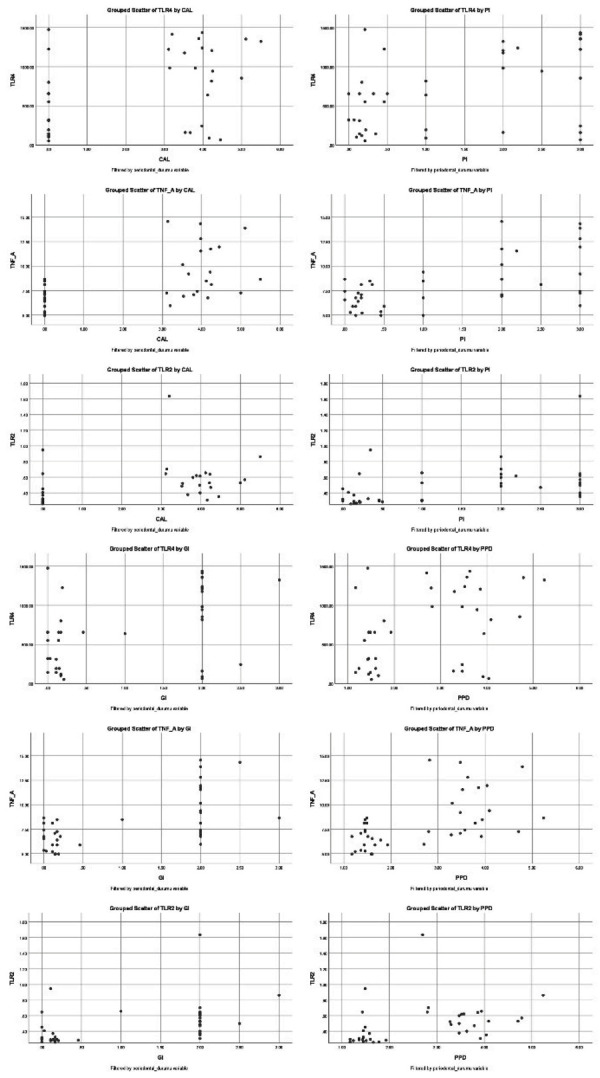
Scatter plot of correlations between clinical periodontal parameters and biomarker levels.

No significant associations were found between clinical parameters and TLR-4 (PI–TLR-4, p = 0.576; GI–TLR-4, p = 0.888; PPD–TLR-4, p = 0.932). Similarly, TNF-α was not significantly associated with TLR-2 (p = 0.941) or TLR-4 (p = 0.922). However, TLR-2 correlated significantly with TLR-4 (r = 0.400, p = 0.011). Figure 3 illustrates scatter plots of the correlations between periodontal parameters and biomarker levels in both groups.

## Discussion

The findings of the present study indicate significant differences in clinical periodontal parameters and inflammatory biomarker levels between periodontally healthy individuals and patients with periodontitis. Following treatment, notable improvements in PPD, CAL, PI, and GI were observed, consistent with prior research demonstrating the effectiveness of NSPT in reducing inflammation and supporting tissue repair.^21^ These results reinforce the established understanding that periodontal therapy reduces clinical disease manifestations by promoting tissue regeneration and modulating inflammatory responses. Furthermore, they highlight the chronic and recurrent nature of periodontal disease, emphasizing that although treatment alleviates symptoms, persistent inflammatory activity requires ongoing management and routine follow-up to sustain periodontal health.^22,23^

Biomarker analyses confirmed the importance of TNF-α, TLR-2, and TLR-4 in the initiation and progression of periodontal disease.^11^ TNF-α, a proinflammatory cytokine secreted primarily by macrophages and monocytes, plays a central role in regulating inflammatory cascades within periodontal tissues.^24^ Elevated TNF-α levels in GCF have been strongly associated with clinical indicators of periodontal disease, including PPD, CAL, and BOP.^15,25^ In the present study, baseline TNF-α concentrations were significantly higher in the periodontitis group but decreased markedly after NSPT. This decline underscores the capacity of periodontal therapy to modulate host inflammatory responses and stabilize periodontal tissues.^26^ Our findings are consistent with previous studies.^21,27^ Ezzat et al.^28^ reported that NSPT reduced GCF TNF-α levels in parallel with clinical improvements. Similarly, Ceylan et al. observed significantly elevated baseline TNF-α levels that decreased after treatment.^25^ Beyond local effects, systemic reductions in TNF-α following periodontal therapy have also been reported,^15,29^ although the present study was restricted to GCF. Collectively, these findings support the value of TNF-α as a biomarker for monitoring periodontal inflammation and assessing therapeutic efficacy.

TLRs, particularly TLR-2 and TLR-4, are key pattern recognition receptors in detecting bacterial pathogens and initiating inflammatory responses in periodontal disease.^11,30,31^ Immunohistochemical studies have shown elevated expression of these receptors in gingival epithelial cells during periodontitis,^11,12^ and their activation is closely linked to the dysbiotic biofilm characteristic of chronic disease.^12^ TLR-2, which primarily recognizes gram-positive bacterial components, is upregulated in periodontitis and promotes cytokine release, including IL-1β and IL-6, contributing to tissue destruction.^32,33^ Conversely, TLR-4, activated mainly by gram-negative lipopolysaccharides, amplifies inflammation and stimulates osteoclastogenesis, accelerating disease progression.^34,35^ In the present study, both TLR-2 and TLR-4 levels were elevated in periodontitis patients compared with healthy individuals, suggesting enhanced innate immune activation in periodontal disease. These findings align with earlier reports and indicate that TLRs may serve as potential markers of dysregulated host–microbe interactions in periodontal inflammation.

Evidence further suggests that NSPT modulates these immune pathways by altering biofilm composition and reducing immunogenic components such as lipopolysaccharides, thereby promoting resolution of inflammation and restoring tissue homeostasis.^36^ In the present study, TLR-4 levels decreased significantly after therapy, whereas TLR-2 remained unchanged, reflecting a differential responsiveness of TLR-mediated pathways at the site-specific level. These results are consistent with an ecological shift from a dysbiotic to a symbiotic microbial community conducive to periodontal health.^14^ Chew et al.^36^ likewise reported reduced TLR-4 activation after NSPT, correlating with clinical improvement. Other studies have shown that periodontal therapy can improve periodontal parameters and normalize soluble TLR-2 levels.^13^ Taken together, these findings suggest that periodontal therapy not only improves clinical outcomes but also regulates host–microbe interactions through TLR-mediated signaling. However, the minimal change in TLR-2 observed in the present study suggests limited utility for early treatment-response monitoring.^13^

Periodontal diagnosis and monitoring rely on clinical indices, imaging, and biofluid biomarkers. While saliva provides a pooled reflection of overall oral status, GCF offers site-specific information about the local inflammatory milieu and can be sampled minimally invasively, making it preferable for monitoring lesion-level changes.^37,38^ Previous studies have reported decreases in TLR-4 activation after nonsurgical therapy in parallel with clinical improvement^36^ and reductions in GCF biomarkers following treatment.^25^ Although TNF-α and/or TLR-4 have been frequently examined in the context of NSPT, changes in TLR-2 and TLR-4 specifically in GCF remain less well characterized.^39,40^ In the present study, only TLR-4 decreased significantly after therapy, while TLR-2 levels remained stable. Thus, although GCF biomarkers may contribute to periodontal diagnosis, their utility for evaluating treatment effectiveness appears limited. The present findings contribute to the literature by providing a simultaneous evaluation of TNF-α, TLR-2, and TLR-4 in GCF before and after NSPT, enabling direct comparison of their responsiveness to therapy. This differential pattern requires confirmation in larger longitudinal studies.

### Limitations

Several methodological limitations should be acknowledged. First, this was a prospective non-randomized interventional study with a healthy comparison group at baseline, but without a randomized parallel control; therefore, causal inference is limited, and regression to the mean or concurrent influences cannot be excluded. Second, although the sample size was sufficient to detect differences in clinical indices, it may not fully represent inter-individual variability in biomarker expression. Larger and more diverse cohorts are needed to confirm reproducibility and generalizability. Third, the follow-up period was relatively short; longer-term studies are required to assess the persistence and clinical significance of changes in TNF-α, TLR-2, and TLR-4. Finally, we focused only on local inflammatory markers in GCF and did not examine systemic biomarkers or microbiological profiles, which would have provided additional context for the observed local responses.

## Conclusion

This study underscores the involvement of pattern recognition receptors and inflammatory mediators in the pathogenesis of periodontitis. Nonsurgical periodontal therapy effectively reduced local inflammation and improved clinical outcomes; however, TLR-4 levels decreased while TLR-2 remained stable, suggesting differential biomarker responsiveness. These findings highlight the importance of monitoring specific inflammatory pathways to better understand host–pathogen interactions in periodontal disease. Future therapeutic strategies should not only target local periodontal inflammation but also address its systemic implications, providing a more comprehensive framework for disease management and prevention.

## Data Availability

The datasets generated and/or analyzed during the current study are not publicly available due to participant privacy considerations. However, in line with ethical approval and data confidentiality agreements, the data may be made available to qualified researchers upon reasonable request from the corresponding author.
